# Characterization of cervical canal and vaginal bacteria in pregnant women with cervical incompetence

**DOI:** 10.3389/fmicb.2022.986326

**Published:** 2022-09-29

**Authors:** Meiguo Sun, Huiwu Geng, Jingjing Bai, Jiahui Feng, Na Xu, Yunlong Liu, Xiaoying Liu, Gang Liu

**Affiliations:** ^1^Department of Obstertrics and Gynecology, The First Affiliated Hospital of Anhui Medical University, Hefei, Anhui, China; ^2^School of Life Sciences, Anhui Medical University, Hefei, Anhui, China; ^3^Translational Research Institute of Henan Provincial People’s Hospital and People’s Hospital of Zhengzhou University, Henan International Joint Laboratory of Non-coding RNA and Metabolism in Cancer, Henan Provincial Key Laboratory of Long Non-coding RNA and Cancer Metabolism, Zhengzhou, Henan, China

**Keywords:** cervical incompetence, bacteria, biomarker, OTUs, vagina and cervical canal

## Abstract

Vaginal and cervical canal bacteria are associated with women’s health and pregnancy outcomes. Here, we compared their composition and characteristics in 37 reproductive-aged Chinese women including 24 pregnant women with cervical incompetence (vaginal and cervical canal bacteria formed Groups A and B, respectively) and 13 healthy pregnant women (vaginal and cervical canal bacteria formed Groups C and D, respectively) using high-throughput sequencing of the V4 region of 16S rRNA gene. The results of alpha and beta diversity analysis, respectively, indicated no statistical differences between Groups A and B (*p* = 0.32, 0.06), nor Groups B and D (*p* = 0.69, 0.74); however, differences were found between Groups C and D (*p* = 0.02, 0.01) and between Groups A and C (*p* = 0.04, 0.02). PLS-DA analysis showed that the individuals from each group were irregularly distributed according to their clade. *Lactobacillus*, *Bifidobacterium* and *Ureaplasma* were the dominant genera in all groups. Phylogenetic Investigation of Communities by Reconstruction of Unobserved States (PICRUSts) analysis identified 31 Kyoto Encyclopedia of Genes and Genomes (KEGG) orthologs associated with the bacterial communities from the four groups, including membrane transport, folding, sorting and degradation, xenobiotics biodegradation and metabolism, and nucleotide metabolism. We further determined relationships between pregnancy outcomes (Apgar scores) and certain bacterial species. A significant positive correlation was found between Apgar scores and *Actinomyces neuii* and *Anoxybacillus flavithermus* in the vagina and cervical canal of pregnant women with cervical incompetence while *Bacteroides plebeius*, *Bifidobacterium pseudopodium* and *Staphylococcus petrasii* in the cervical canal displayed negative correlations with Apgar scores. Moreover, *Clostridium fimetarium*, *Methanobacterium congolense*, *Pseudomonas chlororaphis,* and *Psychrobacter nivimaris* in the vagina were negatively correlated with Apgar scores. These bacteria may serve as potential biomarkers, however, additional research is warranted to verify their role in clinical outcomes.

## Introduction

The female genital tract is home to trillions of bacteria that usually exist in a mutualistic relationship with their host (Younge et al., 2017; Elovitz et al., 2019). Intriguingly, the characteristics of vaginal and cervical canal bacterial communities are even more conspicuous and different from gut bacteria, displaying a high relative abundance of *Lactobacillus* and low bacterial diversity (Kervinen et al., 2019; Villani et al., 2022). Individual differences in the bacterial landscape of the vaginal and cervical canals are dramatically influenced by host genetics and environment, also being impacted by lifestyle factors (Moosa et al., 2020). Recent evidence supports the notion that the bacterial communities of the vagina and cervix are strongly associated with health status, with changes associated with pH, urinary tract infections, sexually transmitted infections including bacterial and viral infections such as HIV (human immunodeficiency virus) along with pregnancy outcomes (Watts et al., 2005; Flaviani et al., 2021; Lykke et al., 2021). Moreover, certain bacterial profiles are associated with adverse obstetric outcomes such as preterm birth, potentially leading to neonatal morbidity and mortality (Elovitz et al., 2019; Torcia, 2019; Tsonis et al., 2020; Tu et al., 2020; Flaviani et al., 2021).

In the female reproductive system, the vagina, cervical canal, fallopian tubes and uterus form the reproductive tract (McDonald, 1980; Chen et al., 2017). In general, the fallopian tubes and uterus are believed to be sterile, which requires the cervix to be a perfect barrier (Chen et al., 2017). The cervix does indeed provide a physical barrier to pathogens, and for example, the fetus is protected from the vaginal pathogens during healthy pregnancies (Hein et al., 2002; Goldenberg, et al., 2008; Aagaard et al., 2014; Liu et al., 2015; Kindinger et al., 2016). However, in some cases bacteria and other pathogens can ascend along the mucosal surfaces of the vagina to the fallopian tubes or the uterus (Liu et al., 2015; Chen et al., 2017; Brown et al., 2019).

Cervical incompetence is defined as the inability of the uterine cervix to retain a pregnancy in the second trimester in the absence of uterine contractions (Boelig et al., 2019). About 1% of females experience painless cervical dilation causing preterm birth in the second trimester, making this one of the most common diseases in reproductive-aged women (Brown et al., 2019; Pilarski et al., 2021). Building evidence over recent years implicates bacteria as the major cause of cervical incompetence, especially the composition and structures of bacteria which are highly correlated with preterm birth (Ravel et al., 2011; Smith and Ravel, 2017; Brown et al., 2019). Nonetheless, the bacterial composition and structures of the vagina and cervical canal in pregnant women suffering from cervical incompetence are yet to be fully characterized (Koedooder et al., 2019; Hao et al., 2021). Focusing on pregnant women with cervical incompetence, this study aimed to expand the current knowledgebase regarding the bacterial profiles of the vagina and cervical canal compared to normal pregnant woman using a high throughput sequencing approach. The results uncover potential new insights into the pathogenesis of cervical incompetence with implications for the prevention and treatment of the associated complications.

## Materials and methods

### Study population

Thirty-seven reproductive-aged Chinese women including 24 pregnant women with cervical incompetence and 13 healthy pregnant women were recruited at the First Affiliated Hospital of Anhui Medical University in 2021, China. We excluded subjects with vaginal inflammation, any acute inflammation, cancer, severe pelvic adhesion, endocrine or autoimmune disorders. Further exclusions were made regarding subjects treated with any vaginal medicine, antibiotics or hormones within one month, other cervical treatment or flushing within one month and sexual intercourse in the prior month (Chen et al., 2017; Tu et al., 2020). This study was approved by the institutional review board of Anhui Medical University (No. PJ2022-09012).

### Sample collection

On the day of the prenatal visit, both vaginal lavage fluid and cervical canal swabs were collected. All collection materials and devices used were strictly sterilized. Cervical canal swabs were used to collect bacteria using a vaginal dilator, while the vaginal samples were performed with 10 ml of vaginal lavage fluid (saline solution). All samples were transferred to the lab within 2 h of collection and stored at −80°C.

### DNA extraction and 16S rRNA ampliconsequencing

One millilitre of sterile phosphate-buffered saline was added to each cervical canal swab and rigorously vortexed for 1 min before 500 μl was collected, centrifuged and disrupted by enzymatic treatment. The vaginal lavage fluids were centrifuged at 2,000 rpm for 15 min. Total DNA of vaginal and cervical canal samples were determined using the Qubit high sensitivity kit follow the manufacturer’s instructions. The V4 variable region of 16S rRNA gene was amplified used the 515F/806R primers (515F: 5′- GTG CCA GCM GCC GCG GTA A -3′; 806R 5′-GGA CTA CHV GGG TWT CTA AT -3′) followed by DNA extraction. The PCR products were purified and then subjected to high-throughput sequencing using the Illumina HiSeq 2,500 at BGI Genomics Co., Ltd. (Shenzhen, China). The raw data of all samples were submitted to the SRA (Sequence Read Archive) under accession numbers SRR19631963 at the NCBI database.

### Bioinformatics, statistical analysis

Raw high-throughput sequencing data provided in FASTA format were subject to pre-processing (quality-trimmed, demultiplexed, and chimera-reduced) using QIIME Version 1.8.0 tools to remove short and poor-quality sequences (Caporaso et al., 2010). The clean reads were clustered to generate Operational Taxonomic Units (OTUs) with 97% similarity cutoff by Vsearch 2.4.2 (Rognes et al., 2016). Representative OUT sequences were given a taxonomic assignment based on the SILVA bacterial database using BLAST Version 2.60 (Altschul et al., 1997). Alpha diversity is a measure of bacteria diversity applicable to a single sample, and it is calculated by Chao1 index in this study. Beta diversity is a measure of similarity or dissimilarity of two communities, which is performed by calculating the UniFrac index by using R (v3.4.1; R Core Team, 2017). Differences between the two populations of the bacterial compositions were analyzed based on Partial Least Squares Discrimination Analysis (PLS-DA) by using the mixOmics package in R (v3.2.1; Rohart et al., 2017). Linear Discriminant Analysis Effect Size (LEfSe) analysis was used between or among groups to determine the differentially abundant taxonomic features by using the non-parametric Kruskal-Wallis rank sum test. Venn analysis diagrams were performed to categorize the core bacteria by using the VennDiagram package in R (v3.2.1; Chen and Boutros, 2011). All statistical analyses and plots were performed by GraphPad Prism v7.0.

## Results

### Sample groups and general sequencing information

We collected and sequenced the 16S rRNA gene from 74 vaginal and cervical canal samples from 37 pregnant women. The vaginal and cervical canal samples from pregnant women with cervical incompetence were designated as Group A (n = 24, sample no. A1-24) and Group B (n = 24, sample no. B1-24), respectively. The samples of the vagina and cervical canal of normal pregnant women were designated as Group C (n = 13, sample no. C1-13) and Group D (n = 13, sample no. D1-13), respectively. After removing low quality reads, a total of 4,825,697 clean reads were retained, which corresponding to 5,537 OTUs, the average reads were 63,496 ± 933 (range, 59,803–64,910 per sample) per sample. An average of 73 OTUs were contained in each sample, which ranged from 18 to 299 OTUs per sample. Samples from Groups A, B, C, and D had 1.06, 3.30, 0.38, and 3.61% unique OTUs with 12.44% of all OTUs common to the four groups (Supplementary Figure S1).

### Alpha and beta diversity

**Figure 1:**
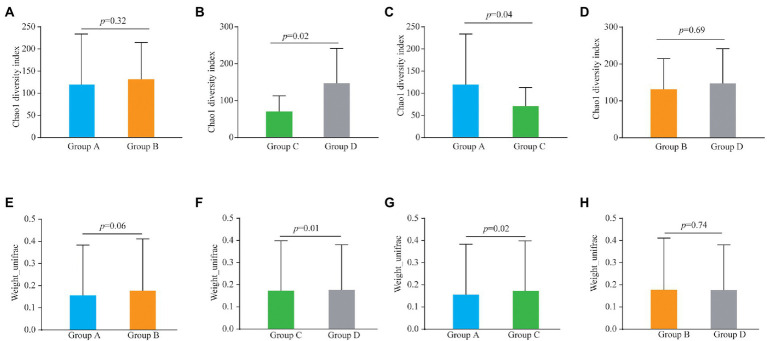
Alpha diversity analysis (Chao1 diversity index) of the bacteria from vaginal and cervical canal samples between Group A and Group B **(A)**, Group C and Group D **(B)**, Group A and Group C **(C)**, and Group C and Group D **(D)**, respectively. Beta diversity analysis of the bacteria from vaginal and cervical canal samples between Group A and Group B **(E)**, Group C and Group D **(F)**, Group A and Group C **(G)**, and Group C and Group D **(H)**, respectively.

Alpha and beta diversities were compared to determine the bacterial composition shift in patients of the four groups. There was no significant differences in Chao1 diversity indexes between Group A and Group B (Figure 1A; *p* = 0.32) and similarly there were no differences between Groups B and D (Figure 1D; *p* = 0.69). However, the Chao1 diversity index showed a significant difference between vaginal samples (Group C) and cervical canal samples (Group D) of healthy pregnant women (Figure 1B; *p* = 0.02), and also between Group A and Group C (Figure 1C; *p* = 0.04).

For the results of beta diversity, there was no significant differences between Groups A and B (Figures1E; *p* = 0.06), and between Groups B and D, respectively (Figure 1H; *p* = 0.74). However, beta diversity was significantly different between Groups C and D (Figure 1F, *p* = 0.01), and beta diversity was also significantly different between Group A and Group C (Figure 1G; *p* = 0.02). PLS-DA analysis results showed that individuals from Group C and D were irregularly distributed according to their clade, and the results of Groups A *vs* C were similar to Groups C *vs* D (Supplementary Figure S2).

### Bacterial community compositions of vaginal and cervical canal

**Figure 2:**
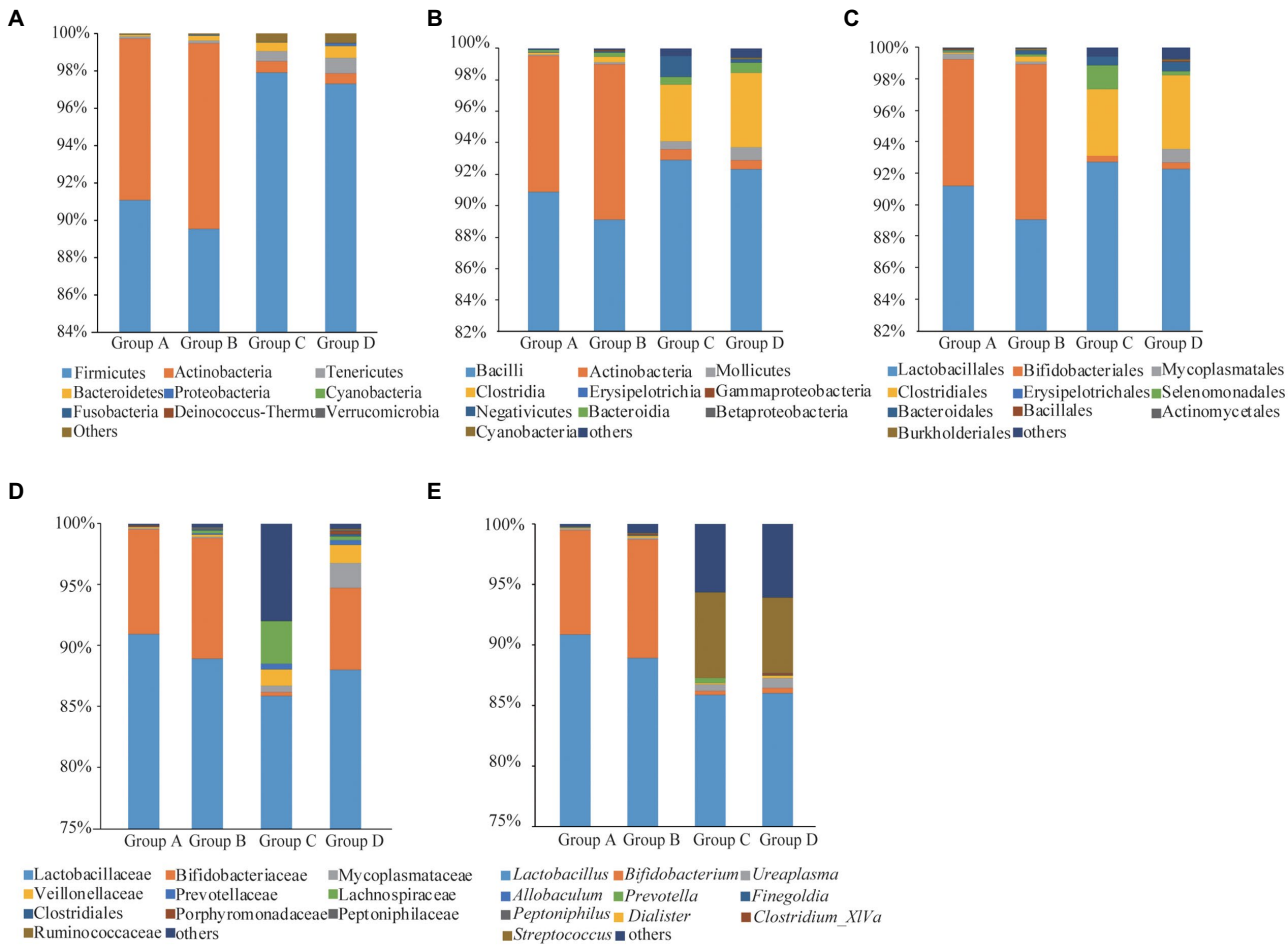
Taxonomic analyses for Groups A, B, C, and D at phyla **(A)**, classes **(B)**, orders **(C)**, families **(D)**, and genera **(E)** levels.

Fourteen phyla, 23 classes, 43 orders, 70 families and 138 genera of bacteria were identified in the four groups. Firmicutes, Actinobacteria and Tenericutes were the dominant bacterial phyla, accounting for 93.97, 4.91, and 0.44% of the OTUs, respectively. Bacteroidetes, Proteobacteria, Cyanobacteria, Fusobacteria, Deinococcus-Thermus, and Verrucomicrobia accounted for less than 0.02% of all bacteria (Figure 2A). Bacilli, Actinobacteria, Mollicutes, Clostridia, Erysipelotrichia, Bacteroidia, Negativicutes, Gammaproteobacteria, Betaproteobacteria and Cyanobacteria were the dominant bacterial classes, Bacilli and Actinobacteria are the dominant bacteria, accounting for 90.89 and 63.48%, respectively (Figure 2B). Lactobacillales, Bifidobacteriales, Mycoplasmatales, Clostridiales, Erysipelotrichales, Selenomonadales, Bacteroidales, Bacillales, Actinomycetales and Burkholderiales were the dominant bacterial orders, and Lactobacillales were the dominant bacteria, accounting for 91.13% (Figure 2C). *Lactobacillaceae*, *Bifidobacteriaceae*, *Mycoplasmataceae*, *Veillonellaceae*, *Prevotellaceae*, *Lachnospiraceae*, *Clostridiales*, *Porphyromonadaceae*, *Peptoniphilaceae* and *Ruminococcaceae* were the dominant bacterial families, *Lactobacillales* were the dominant bacteria, accounting for 88.72% (Figure 2D). The dominant genus was *Lactobacillus* (87.96%; Figure 2E).

### Differences in taxa of the bacteria for vaginal and cervical canal samples

**Figure 3:**
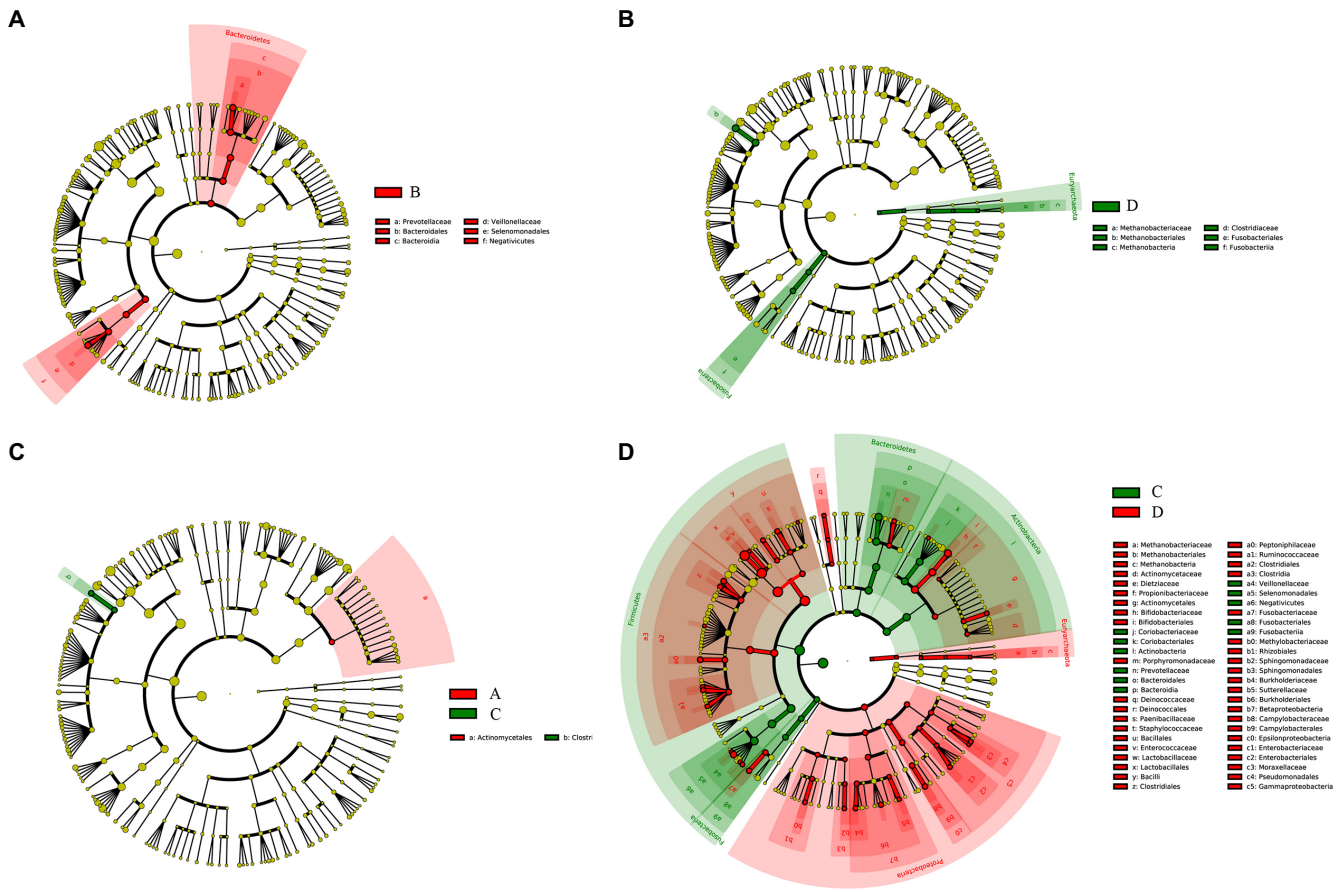
Taxa difference analyses between Group A and Group B **(A)**, Group C and Group D **(B)**, Group A and Group C **(C)**, and Group C and Group D **(D)** by LEfSe analysis, respectively.

**Figure 4:**
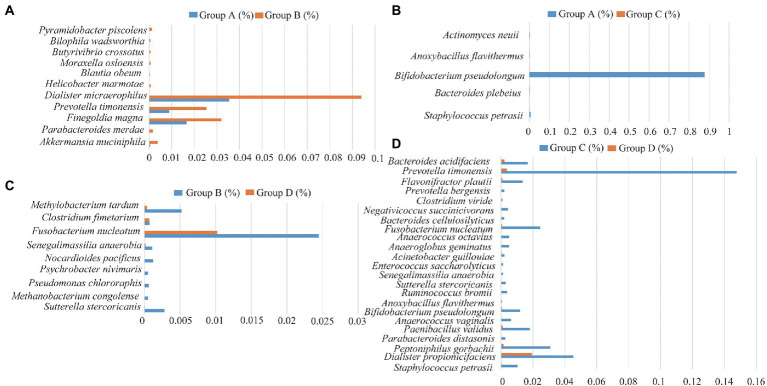
Differences in the species of the bacteria between Group A and Group B **(A)**, Group C and Group D **(B)**, Group A and Group C **(C)**, and Group C and Group D **(D)**, respectively.

The relative abundance of several taxa was compared with each other for the four groups using LEfSe analysis (Figures 3A-D). A total of 42 species of the bacteria were identified from all the vaginal and cervical canal samples of 24 pregnant women with cervical incompetence and 13 healthy pregnant women. There were 11 differences in bacterial abundance found comparing between Groups A and B, the OUT was much lower in Group A than that in Group B (Figure 4A). Five differences were found between the abundance of bacteria in Group A and C, involving much higher abundance of *Actinomyces neuii*, *Anoxybacillus flavithermus*, *Bifidobacterium pseudolongum*, and *Staphylococcus petrasii* in Group A and higher abundance of *Bacteroides plebeius* in Group C (Figure 4B). Nine different species were identified between Groups B and D (Figure 4C), and 18 different species were found between Groups C and D (Figure 4D), respectively. In addition, Group D has significantly higher abundance of OUTs than that in Groups B and C, respectively.

### Functional analysis of KEGG

**Figure 5:**
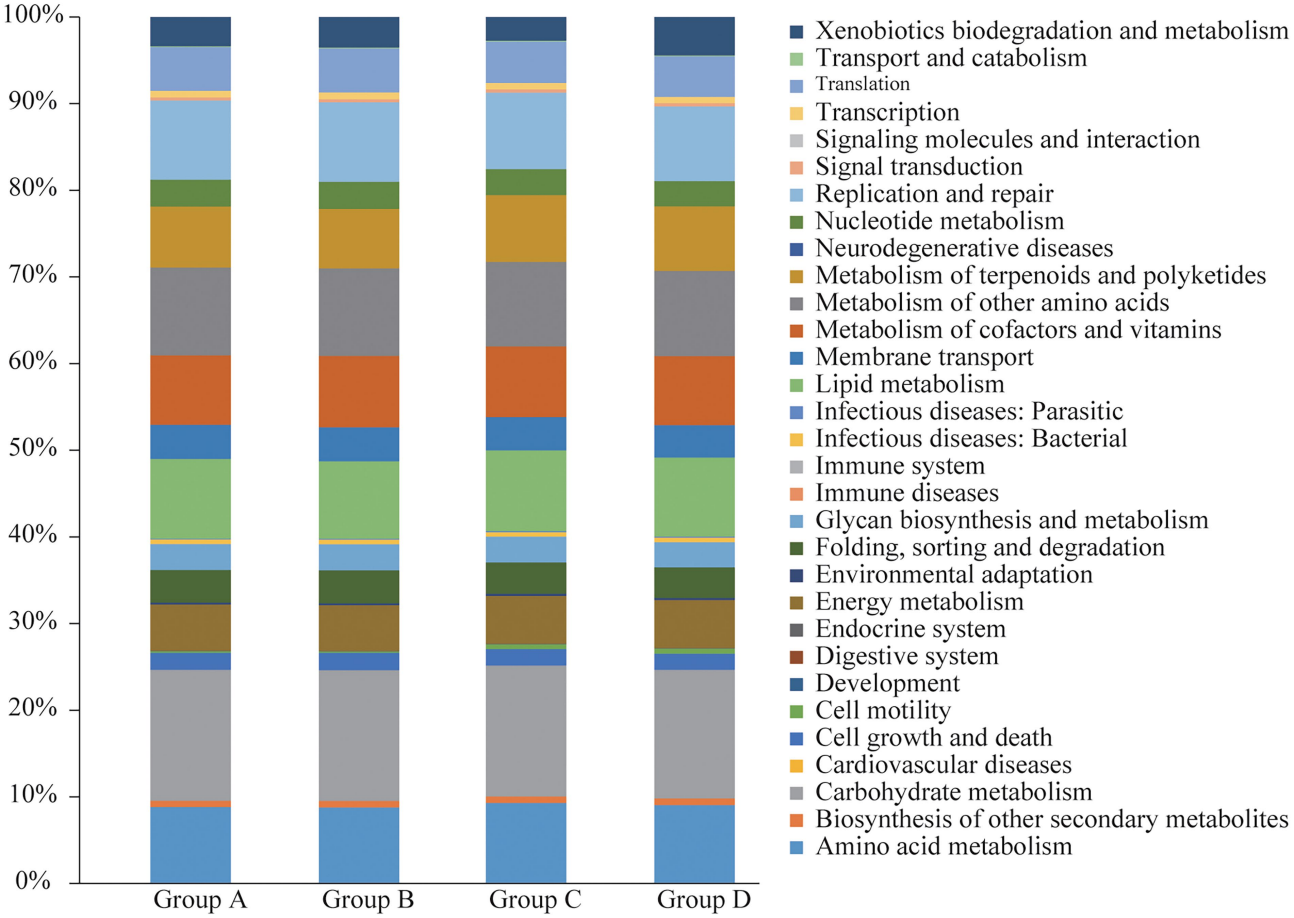
Functional analyses of bacterial composition from Groups A, B, C, and D, respectively.

**Figure 6:**
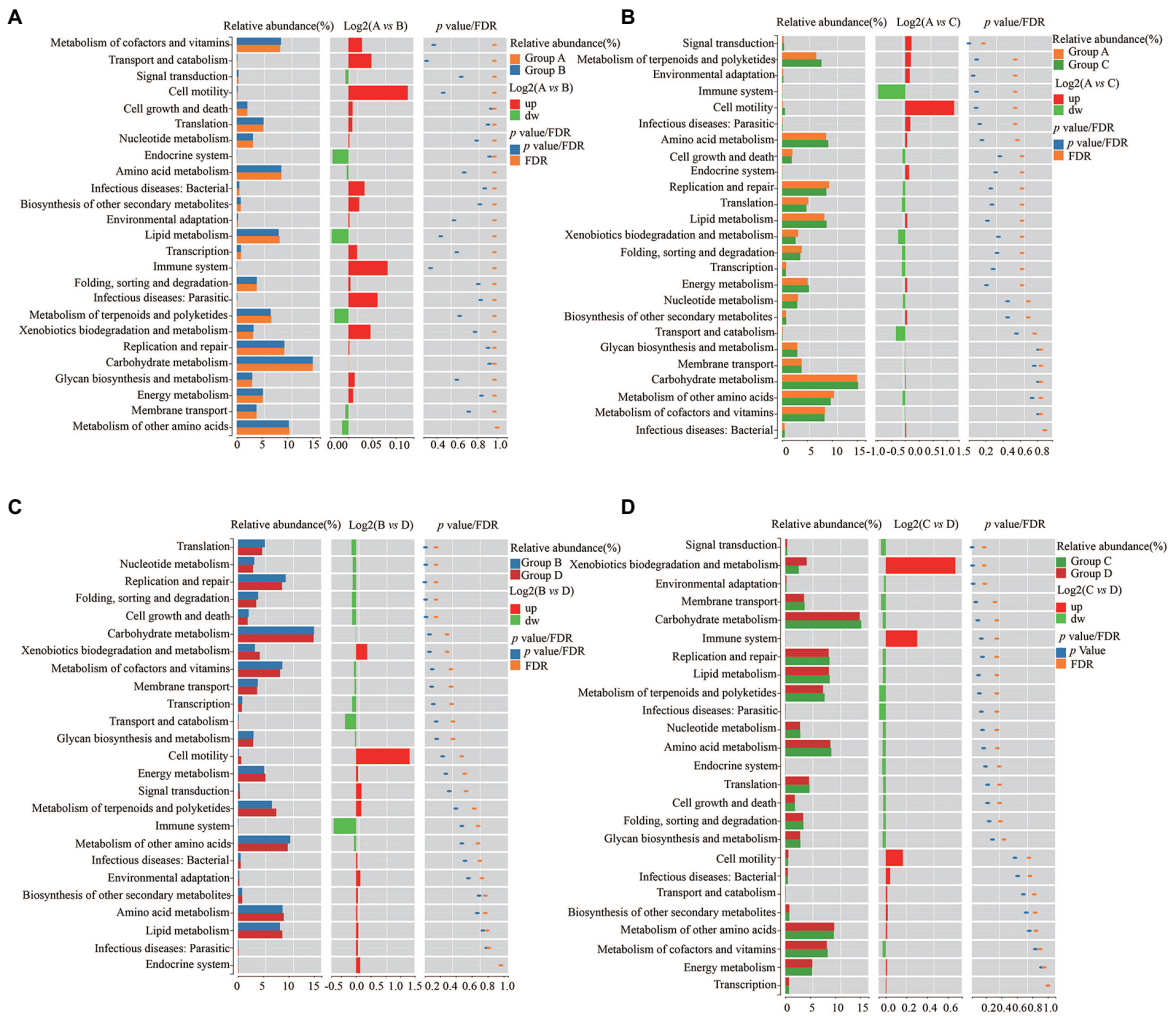
Differences of KEGG bacterial functions between Group A and Group B **(A)**, Group C and Group D **(B)**, Group A and Group C **(C)**, and Group C and Group D **(D)**, respectively.

To further explore the influence of the bacteria on the microenvironment of the vagina and cervical canal, KEGG pathway analyses were conducted. Overall, 31 orthologs were found by PICRUSts predictive exploration tool based on the KEGG database for each group (Figure 5). Half of the KEGG functions were classified into membrane transport, nucleotide metabolism and glycan biosynthesis and metabolism, xenobiotics biodegradation and metabolism, folding, sorting and degradation, etc. We also compared differences in KEGG bacterial function. The digestive system in Group A was significantly different from that in Group B (Figure 6A; *p* = 0.037) and signal transduction was significantly different between Groups A with C (Figure 6B; *p* = 0.006). Moreover, significant differences were found between Groups B with D involving replication and repair (*p* = 0.010), translation (*p* = 0.018), folding, sorting and degradation (*p* = 0.019), nucleotide metabolism (*p* = 0.021), and cell growth and death (Figure 6C; *p* = 0.025). Furthermore, digestive system (*p* = 0.008), signal transduction (*p* = 0.018), immune diseases (*p* = 0.013), environmental adaptation (*p* = 0.031), xenobiotics biodegradation and metabolism (*p* = 0.024) were dramatically different between Groups C and D (Figure 6D).

### Analysis of pregnancy outcomes

We next analyzed pregnancy outcomes of the 37 subjects. The 24 women with cervical incompetence received an intervention during pregnancy involving cervical cerclage. The fetal survival rate was 100% for both groups and there was no significant differences in Apgar scores between healthy pregnant women and those with cervical incompetence (Table 1). However, some complications occurred after the intervention. Five cases of premature rupture of the membrane were diagnosed in women with cervical incompetence, representing 21.74%, higher than that of the normal pregnant women (15.38%; Table 1). Notably, the prematurity rate (defined as birth before 37 weeks) in the cervical incompetence group was 8.70%, which was much higher than that in healthy pregnant women, while one case of abortion was recorded at week 18 of pregnancy (Table 1).

**Table 1:** Analyses of the pregnancy outcomes from normal pregnant women and pregnant women with cervical incompetence.

Indicator types	Group A/B	Group C/D	*Value of p*
Sex rate	Male = 40% Female = 60%	Male = 41.7% Female = 58.3%	
Weight (g)	3037.2 ± 812.3	2465.4 ± 764.2	*p* < 0.01
Survival rate	100%	100%	
Premature rupture of membrane rate	21.74%	15.38%	*p* < 0.01
Prematurity rate	8.70%	0	*p* < 0.05
Abortion individuals	1	1	
Apgar score	9.375 ± 1.317	9.556 ± 1.257	

Group A/B and Group C/D are normal pregnant women and pregnant women with cervical incompetence, respectively.

We also analyzed the relationships between pregnancy outcomes and marker bacteria of the vagina and cervical canal from pregnant women with cervical incompetence receiving cervical cerclage (Supplementary Table 1). Statistical analysis showed that *Actinomyces neuii* and *Anoxybacillus flavithermus* were positively correlated with the pregnancy outcomes (Apgar score). Conversely, there were significant negative correlations between the Apgar score in the cervical canal and *Bacteroides plebeius*, *Bifidobacterium pseudolongum* and *Staphylococcus petrasii*. *Clostridium fimetarium*, *Methanobacterium congolense*, *Pseudomonas Chlororaphis,* and *Psychrobacter nivimaris* was negatively correlated with the Apgar score of pregnancy outcomes in the vagina, while *Allobaculum stercoricanis*, *Fusobacterium nucleatum*, *Methylobacterium tardum*, *Nocardioides pacificus* and *Senegalimassilia anaerobia* were positively correlated.

## Discussion

The human body contains trillions of bacteria inhabiting body surfaces and cavities which can be exchanged with those in the external environment (Ravel et al., 2011). Recent studies have indicated that the bacteria from vaginal and cervical canal play important roles in maintaining a healthy female reproductive system, and moreover, may be mechanistically linked to the pathogenesis of preterm birth (Ansari et al., 2021; Villani et al., 2022). Different bacterial communities or abnormal levels of bacteria populating the vaginal tract and cervical canal during late pregnancy can produce infections or dysfunction, which can adversely affect pregnancy outcomes (Perino et al., 2011; Ravel et al., 2011; Villani et al., 2022). Here, we compared the vaginal and cervical canal bacterial communities between healthy pregnant women and those with cervical incompetence, a condition which may disrupt the distribution of bacteria in the female reproductive tract. Our analysis results for alpha and beta diversity were similar to that of previous studies (Chen et al., 2017).

The bacteria inhabiting the vagina are considered to originate from the upper genital tract (Khan et al., 2016; Tu et al., 2020). In normal pregnant women, the vaginal and cervical canal bacterial communities were different due to the obstruction of the cervix (Chen et al., 2017). In this study we found the bacterial communities of the cervical canal in pregnant women with cervical incompetence were significantly different from that in the normal pregnant women. However, we found no differences in bacterial composition between the vagina and cervical canal of the pregnant women with cervical incompetence, suggesting cervical incompetence promotes bacterial exchange between the vaginal and cervical canal communities. One of the most important risk factors for preterm birth is cervical incompetence in the second trimester of pregnancy. In support of a prior study (Kindinger et al., 2016), we found associations between vaginal and cervical bacteria diversity with preterm delivery. However, larger sample size is needed if we aim to understand the relationships between the vaginal and cervical bacteria diversity and preterm birth.

*Lactobacillus* are the unequivocal dominant bacteria in the vaginal and cervical canal of healthy women. Their production of lactic acid serves to maintain the acidic pH of the vagina and cervical canal, acting as a barrier against pathogens and protects them from genital infection (Petricevic et al., 2014; Chen et al., 2017; Pace et al., 2021). Deficiencies in *Lactobacillus* could disturb the vaginal bacterial balance, resulting in the syndrome of bacterial vaginosis (Petricevic et al., 2014; Lozano et al., 2021). Nonetheless, *Lactobacillus* was reported to remain as the dominant species in pregnant women with cervical incompetence, and no differences were found in the abundance of OTUs compared to healthy pregnant women (Lozano et al., 2021). The vaginal and cervical canal bacteria are dynamic communities owing to physiological, pathological, environmental, and nutritional factors (D’Alterio et al., 2021). Bacterial dysbiosis may contribute to an inflammatory response in the vagina and cervical canal, also predisposing pregnant woman to a higher risk of infection (D’Alterio et al., 2021). Bacterial imbalance of the vaginal and cervical canal is correlated with higher genital pro-inflammatory cytokine concentrations and increased APC (antigen-presenting cell) activation through LPS pathways. Notably, triggering local immunity to increase the concentration of inflammatory factors including TNF-α, NF-κB and COX-2 may affect pregnancy outcomes (Ravel et al., 2011; Smith and Ravel, 2017; D’Alterio et al., 2021).

Similar to the results of previous studies we found differential genera in pregnant women with cervical incompetence including *Staphylococcus*, *Actinomyces*, *Aerococcus*, *Clostridium_sensu_stricto,* and *Anoxybacillus* (Flynn et al., 2013; Chen et al., 2017; Brown et al., 2019; Lozano et al., 2021). We identified Groups A and C -specific shifts in bacterial composition reflected by the enrichment of five species, including *Staphylococcus petrasii*, *Bacteroides plebeius*, *Bifidobacterium pseudolongum*, *Anoxybacillus flavithermus,* and *Actinomyces neuii*. *Bacteroides plebeius* has been found to be more significantly frequent in HPV-positive women, and *Actinomyces neuii* was reported in bacterial vaginosis (Chao et al., 2019; Castro et al., 2019) while the other species are unique to this study. Furthermore, we found the abundances of *Sutterella stercoricanis*, *Methanobacterium congolense*, *Pseudomonas chlororaphis*, *Psychrobacter nivimaris*, *Nocardioides pacificus*, *Senegalimassilia anaerobia*, *Fusobacterium nucleatum*, *Clostridium fimetarium*, *Methylobacterium tardum* were dramatically different between Groups B and D. Collectively, these results suggest that these species represent “biomarker” bacteria for differences between healthy pregnant women and those with cervical incompetence (Agarwal et al., 2020).

As an important potential anaerobic pathogen and Gram-negative bacterium, *Fusobacterium nucleatum* colonizes the female reproductive tract, digestive tract and oral cavity (Agarwal et al., 2020). This species has been associated with numerous human diseases, including oral infections, colorectal cancer, respiratory tract infections, and Alzheimer’s disease (Allen-Vercoe et al., 2011; Liu et al., 2014; Han, 2015; Agarwal et al., 2020). *Fusobacterium nucleatum* was also detected in a wide spectrum of fetal membranes, amniotic fluid, neonatal gastric aspirates, chorioamnionitis, and was frequently detected in the cord blood and amniotic fluid of premature infants, being associated with intrauterine infection and preterm birth (Han et al., 2010; Wang et al., 2013; Han, 2015). Additional research also indicated that the mother’s subgingival plaque in oral cavity may transfer *Fusobacterium nucleatum* to the placenta or fetus during pregnancy period, leading to acute inflammation, even leading to fetal stillbirth (Han, 2011; Wang et al., 2013). However, further validation is still needed to confirm the utility of the biomarker bacteria identified here and in other studies.

Preterm birth occurs in 5–20% of pregnant women and is defined as delivery before 37 weeks of gestation. Preterm birth is influenced by maternal, fetal and environmental factors, and is associated with a high risk of neonatal morbidity and mortality (Terzic et al., 2021). Some researches have indicated that cervical incompetence is related to preterm birth, although other potential risk factors can influence the incidence of preterm delivery, including diabetes, hypertension, thyroid disease, chronic renal disease and tobacco smoking (Guerra et al., 2006; Goldenberg et al., 2008; Vogel et al., 2018; Miklavcic et al., 2021). In addition, pre-pregnancy body mass index, advanced maternal age (more than 35 years old), endometriosis, polycystic ovary syndrome, and pre-pregnancy diabetes are also considered possible risk factors to preterm birth (Molin, 1993; Vyas et al., 2006; Ciancimino et al., 2014; Miklavcic et al., 2021; Pilarski et al., 2021). Other studies suggest that bacterial vaginal infection and maternal periodontal diseases increase the risk of preterm birth, with bacterial transfer from the vagina to the uterus through the cervix (D’Alterio et al., 2021; Terzic et al., 2021). Pregnant women with intrauterine infection could also spread bacteria to the amniotic cavity to cause preterm birth (Angioni et al., 2015). Some studies reported that periodontal diseases of pregnant women were associated with preterm birth, the proposed reason being gingival bacterium transfer to the uterine cavity and placenta *via* the bloodstream, resulting in an intra-amniotic infection (Offenbacher et al., 2006; Ren and Du, 2017).

In summary, we investigated the bacterial compositions and structures of the vagina and cervical canal from healthy pregnant women and pregnant women with cervical incompetence by high-throughput sequencing technology. The overall bacteria were not significantly different in *Lactobacillus* in the vaginal and cervical canal of healthy pregnant women and pregnant women with cervical incompetence. However, the alpha and beta diversities were significantly different between vaginal samples and cervical canal samples of normal pregnant women. We also found differences in the bacterial communities of cervical canal samples between normal pregnant women and those with cervical incompetence. Several specific bacteria were identified, suggesting that these bacteria may act as potential biomarkers. However, we must also acknowledge certain limitations regarding our study. First, the sample size is relatively small. Second, further investigations are required to elaborate the functional consequences of the “biomarker” bacteria. Third, more experimental technologies are necessary to reveal the correlation between bacteria and clinical features and elucidate the molecular mechanism.

## Data availability statement

The datasets presented in this study can be found in online repositories. The names of the repository/repositories and The datasets presented in this study can be found in online repositories. The names of the repository/repositories and accession number(s) can be found in the article/Supplementary material.

## Ethics statement

The studies involving human participants were reviewed and approved by Anhui Medical University. Written informed consent for participation was not required for this study in accordance with the national legislation and the institutional requirements.

## Author contributions

MS, GL and XL designed the experiments of this manuscript. MS, HG, JF, and GL performed the experiments. MS, NX, YL, and GL analyzed the data. MS, YL, and GL wrote the manuscript. All authors contributed to the article and approved the submitted version.

## Funding

This research was supported by the National Natural Science Foundation of China (grant nos. 31702030 and 81772908) and the Natural Science Foundation for the Higher Education Institutions of Anhui Province of China (grant no. KJ2021A0246).

## Conflict of interest

The authors declare that the research was conducted in the absence of any commercial or financial relationships that could be construed as a potential conflict of interest.

## Publisher’s note

All claims expressed in this article are solely those of the authors and do not necessarily represent those of their affiliated organizations, or those of the publisher, the editors and the reviewers. Any product that may be evaluated in this article, or claim that may be made by its manufacturer, is not guaranteed or endorsed by the publisher.
