# Supplementary material for: Characterization of cervical canal and vaginal bacteria in pregnant women with cervical incompetence
Source: Front Microbiol. 2022 Sep 29;13:986326. doi: 10.3389/fmicb.2022.986326 (PMC9556877; doi:10.3389/fmicb.2022.986326)
Supplement: Supplementary file 1 [file Data_Sheet_1.DOCX]

**Supplementary Figures**


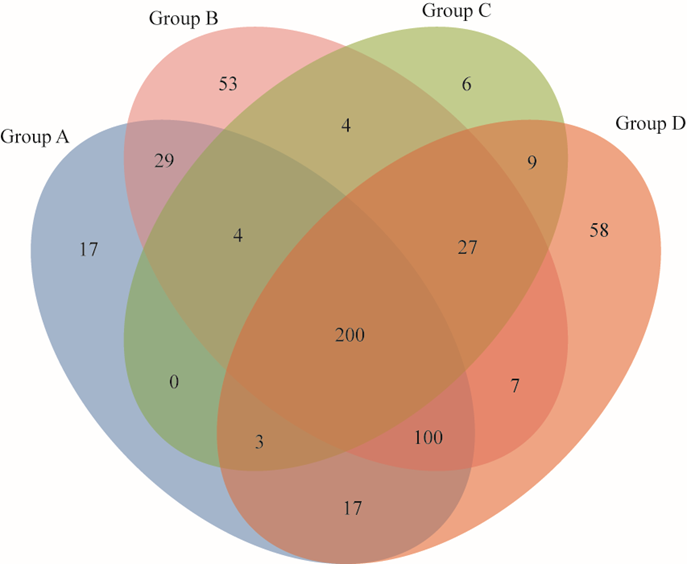


Supplementary Figure 1. Venn diagrams illustrating the numbers of operational taxonomic units (OTUs) for the bacteria of Groups A, B, C, and D.


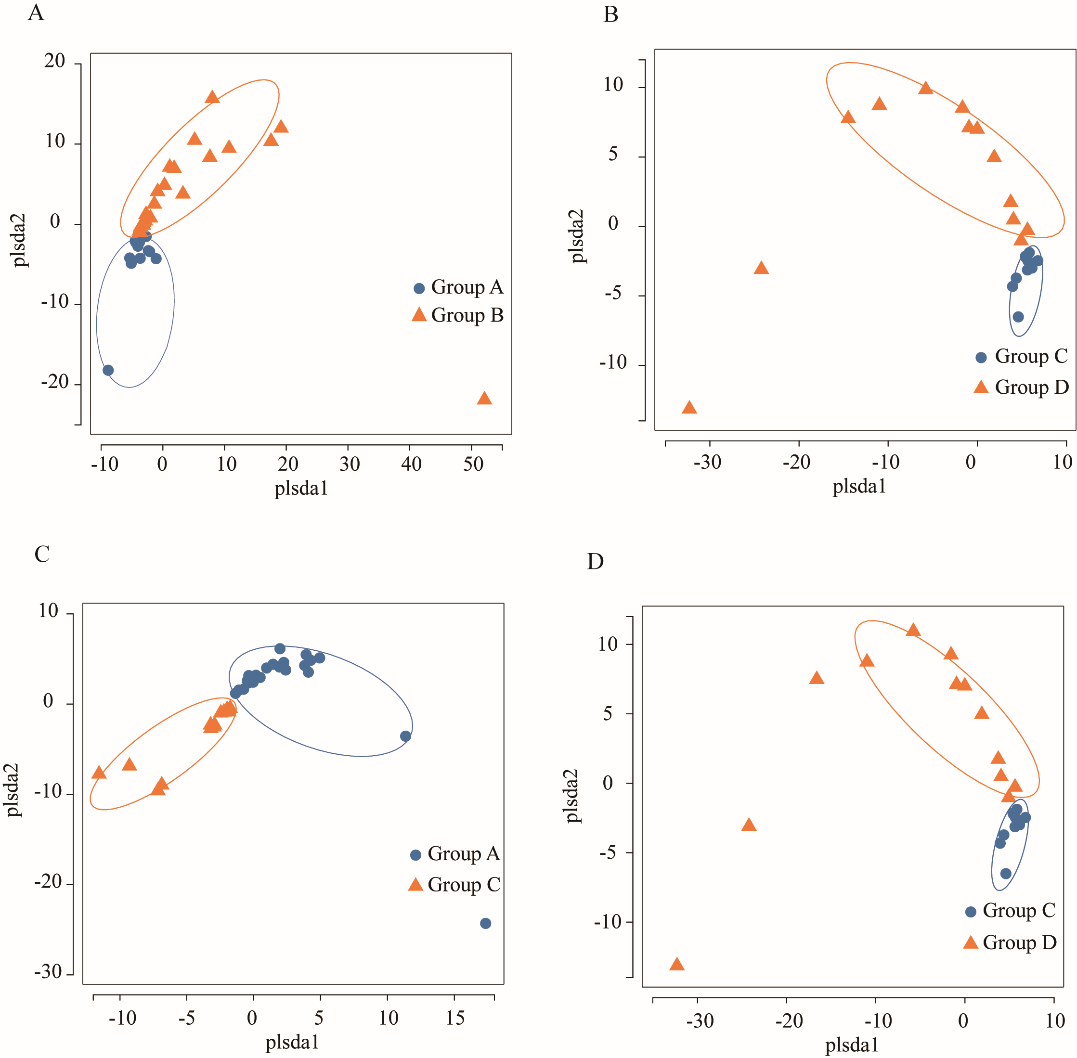


Supplementary Figure 2. The characteristics of the bacteria from vaginal and cervical canal samples between Group A and Group B (A), Group C and Group D (B), Group A and Group C (C), and Group C and Group D (D) by PLS-DA analysis.

**Supplementary table**

Supplementary Table 1. Analysis of the relationships between Apgar score of pregnancy outcomes and some marker species.

| Type | Species | Regression equation | R-value |
| --- | --- | --- | --- |
| Cervical canal (Groups A *vs* C) | *Actinomyces neuii* | y = 65.876x + 9.386 | 0.134 |
|  | *Anoxybacillus flavithermus* | y = 52.700x + 9.391 | 0.120 |
|  | *Bacteroides plebeius* | y = -0.021x + 9.458 | -0.070 |
|  | *Bifidobacterium pseudolongum* | y = -0.023x + 9.158 | -0.069 |
|  | *Staphylococcus petrasii* | y = -70.904x + 9.546 | -0.174 |
| Vagina (Groups B *vs* D) | *Allobaculum stercoricanis* | y = 7.413x + 9.424 | 0.046 |
|  | *Clostridium fimetarium* | y = -239.920x + 9.638 | -0.576 |
|  | *Fusobacterium nucleatum* | y = 1.950x + 9.399 | 0.085 |
|  | *Methanobacterium congolense* | y = -325.200x + 9.501 | -0.168 |
|  | *Methylobacterium tardum* | y = 13.200x + 9.402 | 0.082 |
|  | *Nocardioides pacificus* | y = 129.290x + 9.391 | 0.127 |
|  | *Pseudomonas chlororaphis* | y = -417.820x + 9.571 | -0.285 |
|  | *Senegalimassilia anaerobia* | y = 67.382x + 9.393 | 0.074 |
|  | *Psychrobacter nivimaris* | y = -418.800x + 9.461 | -0.291 |
